# Efficient and Rapid Photocatalytic Degradation of Methyl Orange Dye Using Al/ZnO Nanoparticles

**DOI:** 10.3390/nano11041059

**Published:** 2021-04-20

**Authors:** Piangjai Peerakiatkhajohn, Teera Butburee, Jung-Hoon Sul, Supphasin Thaweesak, Jung-Ho Yun

**Affiliations:** 1 Faculty of Environment and Resource Studies, Mahidol University, Nakhon Pathom 73170, Thailand; piangjai.pee@mahidol.ac.th; 2 National Nanotechnology Center, National Science and Technology Development Agency, 111 Thailand Science Park, Pathum Thani 12120, Thailand; teera.but@nanotec.or.th; 3 School of Engineering and Technology, Central Queensland University, Mackay, QLD 4740, Australia; j.sul@cqu.edu.au; 4 Department of Chemical Engineering, Faculty of Engineering, Burapha University, Chon Buri 20131, Thailand; 5 Nanomaterials Centre, School of Chemical Engineering and Australian Institute for Bioengineering and Nanotechnology (AIBN), The University of Queensland, St Lucia, QLD 4123, Australia

**Keywords:** photocatalytic degradation, ZnO nanoparticles, Al-doped, sol-gel, methyl orange

## Abstract

ZnO and Aluminum doped ZnO nanoparticles (Al/ZnO NPs) were successfully synthesized by the sol-gel method. Together with the effect of calcination temperatures (200, 300 and 400 °C) and Al dosage (1%, 3%, 5% and 10%) on structural, morphological and optical properties of Al/ZnO NPs, their photocatalytic degradation of methyl orange (MO) dye was investigated. The calcination temperatures at 200, 300 and 400 °C in forming structure of ZnO NPs led to spherical nanoparticle, nanorod and nanoflake structures with a well-crystalline hexagonal wurtzite, respectively. The ZnO NPs calcined at 200 °C exhibited the highest specific surface area and light absorption property, leading to the MO removal efficiency of 80% after 4 h under the Ultraviolet (UV) light irradiation. The MO removal efficiency was approximately two times higher than the nanoparticles calcined at 400 °C. Furthermore, the 5% Al/ZnO NPs exhibited superior MO removal efficiency of 99% in only 40 min which was approximately 20 times enhancement in photocatalytic activity compared to pristine ZnO under the visible light irradiation. This high degradation performance was attributed to the extended light absorption, narrowed band gap and effective suppression of electron–hole recombination through an addition of Al metal.

## 1. Introduction

Azo dyes, especially methyl orange, are mainly used in the textile, paper, synthetic leather and food industry due to high chemical stability [1,2]. Effluents containing dyes discharged into surface and ground water can lead to very serious environmental problems such as mutagenic and carcinogenic to humans and aquatic organisms [3,4]. To reduce the harmful effects of these pollutants on the environment and follow stringent environmental regulations, the research on treatment of azo dyes in wastewater has thus been intensified. Various traditional treatment methods for removing azo dyes in wastewater such as physical [5,6], chemical [7,8] and biological [9,10] methods have been explored. Although these methods are effective in removing organics contaminants, they cannot completely destroy dye molecules. For many cases, the dyes are just adsorbed on the adsorbent. Moreover, high operational cost and sludge production limit their practical applications. Thus, it is necessary to develop alternative approaches which are inexpensive, efficient and environmentally-friendly processes for the complete degradation of pollutants. Advanced oxidation process (AOP) by photocatalysis has been considered as one of the most promising candidates in degrading complex organic compounds in polluted water [7,11]. Photocatalysis can be conveniently applied for degradation of dye pollutants without secondary pollution owning to the complete mineralization of organic dyes into H_2_O, CO_2_ and mineral acids [12]. Metal semiconductor materials, such as TiO_2_ [13,14], ZnO [15,16], Fe_2_O_3_ [17,18,19] and Cu_2_O [20,21,22], are used as a photocatalyst.

Among various semiconductors, ZnO exhibits a great potential with high efficiency in degrading some organic dyes, due to low cost, good chemical stability and high photosensitivity [23,24]. ZnO is an n-type semiconductor, with a wide bandgap of approximately 3.37 eV and a high excitation binding energy of 60 mV, which produces electron–hole pairs under light irradiation. The excited electron and hole can reduce and oxidize the organic contaminants completely into their respective end products. Versatile material features of ZnO can be further enhanced through various synthesis techniques such as hydrothermal synthesis [25,26], homogeneous precipitation [27,28], electrodeposition [29,30], solvothermal [31,32] and sol-gel methods [33,34,35]. However, its large bandgap limits the utilization of solar light to only UV region [36,37], which is a critical drawback in employing ZnO as a photocatalyst. Therefore, it has become an important issue to extend the optical absorption of ZnO from the UV region to visible region for photocatalytic applications. Several methods have been proposed to improve properties of ZnO by controlling the size and morphology of particles [38,39], doping with metals and nonmetals [40,41,42,43] and coupling with other semiconductor catalysts [44,45,46]. The surface defects by introducing metal elements have been reported to play an important role in improving photocatalytic activities over a wide range of wavelengths by reducing the bandgap energy. Aluminum (Al), which is superabundant, inexpensive and non-toxic, is a suitable impurity dopant for photocatalytic activity. Particularly, doping of ZnO with Al has shown the improvement of photocatalytic activity [47].

In this work, we prepared ZnO and Al/ZnO photocatalytic NPs with various amount of Al dopant by a facile sol-gel technique and studied their photocatalytic activity of MO dyes. Importantly, the effects of calcination temperatures and Al doping amount were investigated through the comprehensive physicochemical characterizations and their photocatalytic activity measurement, thereby proposing a possible photocatalysis mechanism in decomposing MO dyes through the Al/ZnO NP system.

## 2. Materials and Methods 

### 2.1. Preparation ZnO and Al/ZnO Photocatalysts 

ZnO and Al/ZnO were synthesized by the conventional sol-gel method. Typically, zinc acetate (Zn (CH_3_COO)_2_·2H_2_O) 0.15 M was dissolved in methanol and stirred for 30 min to obtain a ZnO solution. To obtain Al/ZnO, 0.15 M of aqueous aluminum nitrate (Al(NO_3_)_3_) as a source of aluminum was prepared, drop-wisely added to the ZnO solution with different amounts (1, 3, 5 and 10 mol%) and continuously stirred for 90 min. The pH of the mixture solutions was adjusted to 10 by 1.5 M sodium hydroxide (NaOH), and a white colloid was obtained. Then, the white colloid sol was aged to form a gel and stirred for 60 min. These samples were then centrifuged and dried at 70 °C. The fine white ZnO powder obtained after grinding was subsequently calcined in air at a ramp rate of 2 °C min^−1^ to the desired temperatures (200, 300 and 400 °C) and maintained for 2 h. Meanwhile, Al/ZnO NPs were obtained by the thermal treatment of calcination temperature at 200 °C for 2 h with the ramping rate 2 °C/min. 

### 2.2. Characterization

The as-prepared photocatalysts were characterized by X-ray diffraction (XRD; Bruker, D2 Phaser) using the Cu Kα1 radiation in a range of 20–70°. The surface morphology and elemental analysis of the samples were examined using field emission scanning electron microscopy (FE-SEM, JEOL 2100Plus, Tokyo, Japan) and energy-dispersive X-ray spectroscope (EDX; FEI Model Versa 3D). Specific surface areas were determined by N_2_ adsorption (2800P V-Sorb) and calculated using Brunauer–Emmett–Teller (BET) theory. Furthermore, the light absorption spectrum and photocatalytic activity were investigated by a UV–Vis spectrophotometer (JASCO V-630, Tokyo, Japan).

### 2.3. Photocatalytic Measurement 

The photocatalytic performance of the nanoparticle photocatalysts was evaluated by testing the decolorization of a synthetic MO dye solution (Sigma-Aldrich, St. Louis, MO, USA, 85% purity) with the initial concentration of 50 mg/L under visible light irradiation. In the photocatalysis, 30 mg of photocatalyst was added to 30 mL of aqueous MO dye solution under continuous stirring. The dye solution with the photocatalyst was stirred and kept in the dark to reach adsorption–desorption equilibrium before irradiation. The distance between the reactor and the lamp was kept at 10 cm. The photocatalytic performance of ZnO samples with various calcination temperatures were investigated under 10 W UV lamp irradiation (Philips T8, λ = 365 nm, intensity 2.3 mW/cm^2^). Meanwhile, the photocatalytic performance of Al/ZnO photocatalyst samples were evaluated under 10 W visible Florescence lamp (Philips T, λ = 604 nm, intensity 4.5 mW/cm^2^). The dye solution sample was collected at a regular interval of 5 min. The collected sample was immediately centrifuged at 8200 rpm for 10 min to separate the photocatalyst particles. Finally, the supernatant liquid was analyzed by the UV–Vis spectrophotometer to obtain the absorbance for quantifying the concentration of MO (the maximum absorption wavelength of the dye at 460 nm). The absorption spectra of the MO dye solutions were measured at a regular interval of 5 min. The rate of degradation was reported by calculating the percentage of the concentration of the dye remained over the initial dye concentration, obtained from the absorbance standard curve. The degradation performance can be evaluated by considering the following Equations (1) and (2):

Degradation (%) = [(C_0_ − C_t_)/C_0_)] × 100 (1)

where C_0_ and C_t_ are the initial and final concentrations of methyl orange (mg/L) at reaction time (t), respectively.

ln (C_0_/C_t_) = kt (2)

where C_0_/C_t_ is the normalized MO concentration, k is the first-order rate constant and t is the reaction time.

## 3. Results and Discussion

### 3.1. Effect of Calcination Temperature on the Properties of ZnO NPs

**Figure 1:**
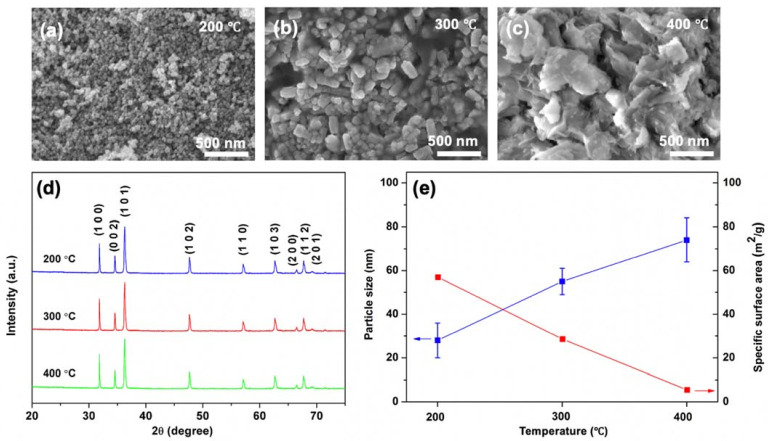
FE-SEM micrographs showing the morphology of of ZnO NPs derived from various calcination temperatures at: (**a**) 200 °C; (**b**) 300 °C; and (**c**) 400 °C. (**d**) X-ray diffraction patterns of the ZnO NPs after calcination at the corresponding temperatures. (**e**) Specific surface area and particle size with deviation of ZnO NPs calcined at different temperatures.

The influence of various calcination temperatures, at 200, 300 and 400 °C, on the crystallinity, morphological, optical properties and photocatalytic activity of MO dye was investigated for ZnO NPs. FE-SEM images of the prepared ZnO NPs that were calcined at 200, 300 and 400 °C are shown in Figure 1a–c, respectively. Calcination at 200 °C led to the formation of compact structures composing highly uniform spherical ZnO NPs. It reveals that the increase in calcined temperature leads to the increased particle size and agglomeration of the as prepared samples. The ZnO NPs calcined at 300 and 400 °C exhibited well defined nanorods (NRs) and nanoflakes shape (NFs), respectively. Under higher reaction temperature, the formation of these ZnO NPs with less densely packed shapes such as NRs and NFs can be due to the enhanced frequency of collisions among nucleation atoms during synthesis process, which quickly increases surface mobility and inhibits aggregation of the formed particles [48]. The XRD patterns of the synthesized ZnO NPs with different calcination temperatures are depicted in Figure 1d. ZnO NPs exhibited a well-crystalline hexagonal wurtzite ZnO crystal structure (JCPDS card No. 36-1451). All the samples of ZnO NPs featured diffraction peaks at 31.8°, 34.51°, 36.37°, 47.59°, 56.64°, 62.86°, 66.40°, 67.91° and 69.15°, corresponding to (100), (002), (101), (102), (110), (103), (200), (112) and (201) planes of ZnO, respectively. It can be noticed that the full width at half maximum (FWHM) of (101) peaks of ZnO NPs at 200, 300 and 400 °C are 0.48°, 0.39° and 0.23°, respectively where the higher FWHM indicates the smaller particle size of ZnO NPs in accordance with the previous report [49]. In addition, the average grain size of ZnO NPs was determined from the XRD patterns using the following Debye–Scherrer equation (Equation (3)) [50]

(3) D=kλ/βcosθ

where D is the crystallite size (nm), λ is the wavelength of the X-ray radiation (Cu-*Kα* = 0.1541 nm), k is the constant coefficient for the reciprocal lattice point about 0.94, β is the full width at half maximum of the intense and broad peaks and θ is the Bragg’s angle. 

The average particle size and its standard deviation and specific surface area of ZnfO NPs are shown in Figure 1e. The results show that the particle size of ZnO NPs increased with increased calcination temperatures. The ZnO NPs calcined at 200, 300 and 400 °C exhibited the average particle sizes of 28 ± 8, 55 ± 6 and 71 ± 10 nm, respectively. In addition, the specific surface area decreased in contrast to increasing the calcination temperature. The ZnO NPs calcined at 200 °C showed the highest specific surface area of 54 m^2^/g, while ZnO NPs calcined at 300 and 400 °C revealed lower specific surface area of 27 m^2^/g and 5 m^2^/g, respectively. The results indicate that a decrease in grain size is associated with an increase in the specific surface area of ZnO NPs calcined at different temperatures. 

**Figure 2:**
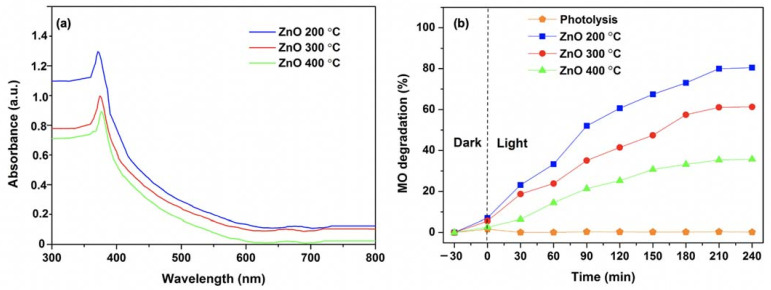
(**a**) UV−Vis spectra; and (**b**) MO degradation efficiency of ZnO NPs at different calcination temperatures (200, 300 and 400 °C) under UV light irradiation.

The UV–Vis absorption spectra of ZnO NPs at the different calcination temperatures are shown in Figure 2a. It reveals that all the ZnO NPs have the weak and broad absorption in visible region between 400 and 800 nm with the sharp absorption peaks at 371, 375 and 377 nm for 200, 300 and 400 °C, respectively, corresponding to the characteristic band of ZnO NPs. The sharp absorption peak of ZnO NPs is attributed to the transition of electron from the valence band to the conduction band, confirming the direct band gap absorption of ZnO NPs [51,52]. The calculated energy band gaps for the ZnO NPs calcined at 200, 300 and 400 °C are 3.34, 3.30 and 3.28 eV, respectively. Additionally, no other peak was observed which confirmed the high purity of the ZnO NPs. Notably, it can be noticed that the lower calcination temperatures caused a blue shift in UV–Vis spectra with the wider bandgap energy. This could be attributed to the quantum confinement effect by reducing particle size at low calcination temperatures [53,54]. Furthermore, the shifting of absorption peaks relates to the existence of oxygen vacancies and the disordered crystal structure of ZnO NPs [55,56]. Typically, the widened bandgap of ZnO NPs could significantly extend the UV response, resulting in the improved photocatalytic performance in the UV region. Thus, the widening bandgap energy with high absorption intensity of the ZnO NPs calcined at 200 °C is beneficial for the photocatalytic activity of MO under the UV light irradiation. The photocatalytic performance of the ZnO samples with the different calcination temperatures for the MO solution is illustrated in Figure 2b. Prior to the photocatalytic test, the photocatalyst and MO dye solution was kept in the dark for 30 min to obtain the equilibrium adsorption of the photocatalyst. In addition, the photolysis of MO dye under UV irradiation was carried out. The result shows that the MO dye was quite stable under UV light irradiation. The ZnO NPs calcined at 400 °C shows the lowest photocatalytic activity in MO degradation that removed only about 35% of MO after 240 min under UV light irradiation. In contrast, the ZnO NPs samples prepared at the lower calcination temperatures show better activity in MO degradation. Among these results, the ZnO NPs calcined at 200 °C exhibits the highest activity and about 80% of MO was decomposed after 240 min under UV light irradiation. The result corresponds to the higher specific surface area of ZnO NPs calcined at 200 °C compared to that of ZnO NPs calcined at 300 and 400 °C.

It can be noticed that the photocatalytic activities for the ZnO NPs samples were gradually decreased with elevated calcination temperatures. This tendency clearly shows that the calcination temperature significantly affects the photocatalytic behaviors of ZnO NPs. This can be due to a difference of specific surface area and a change of light absorption property, induced by different morphologies and particles sizes of ZnO NPs, resulting from the varying calcination temperatures. The photocatalytic performance of the smaller sized ZnO NPs calcinated at 200 °C was greatly improved with its higher specific area and light absorption, as shown in Figure 2a. The size, uniformity and morphology of the nanoparticles in ZnO NPs are advantageous to their photocatalytic activity, as compared to ZnO NRs and ZnO NFs. In addition, the previous studies revealed that the concentration of oxygen vacancies depends on calcination temperature. The oxygen vacancies are higher at low calcination temperature compared to that of high calcination temperature [56,57]. Consequently, the concentration of oxygen vacancies in the ZnO NPs samples and size of the ZnO NPs obtained at the different calcination temperatures changed light absorption and photocatalytic activity. 

### 3.2. Effects of Al Dopant on the Properties of ZnO NPs 

**Figure 3:**
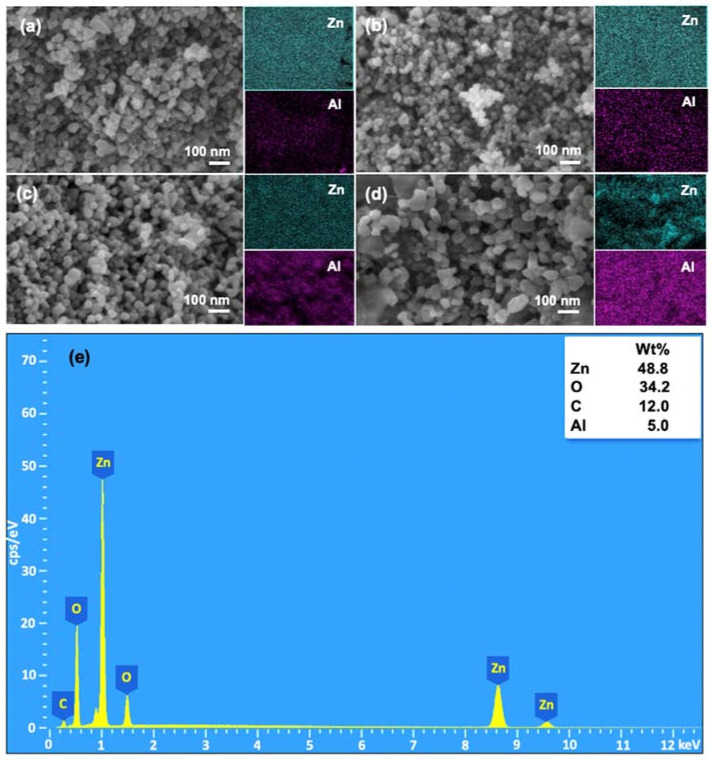
FE-SEM and elemental mapping images of: (**a**) 1% Al/ZnO; (**b**) 3% Al/ZnO; (**c**) 5% Al/ZnO; and (**d**) 10% Al/ZnO photocatalysts. (**e**) The EDX spectrum of the 5% Al/ZnO.

The morphology of Al/ZnO NPs calcined at 200 °C with various Al concentrations is shown in Figure 3a–d. The FE-SEM images and elemental mapping revealed the compact structure of highly uniform spherical particles of the Al/ZnO photocatalysts, regardless of the concentration of Al. However, it was observed that there are differences in particle aggregation of Al/ZnO photocatalysts with the change of aluminum content. With an excessive amount of Al at 10% concentration, the structure of nanoparticles is turned into a rod-like structure, as shown in Figure 3d. This might be because an incorporation of Al^3+^ into ZnO matrix can produce planar defects along plane, which may raise the surface energy and lead to quick anisotropic growth along different direction without affecting the intrinsic polarity of nanostructures [58]. In addition, the morphological change from the doping could be attributed to an increase in charges when Zn^2+^ sites are replaced by Al^3+^ into the lattice [59]. In other words, the positive charge increases plane growth along one direction. Elemental mapping depicts the uniform distribution of Zn and Al atoms in the particle. The EDX spectra of the 5% Al/ZnO photocatalyst is shown in Figure 3e, which exhibits the presence of Zn and Al atoms, as well as confirms that the sample is free of other impurities [60]. Furthermore, the specific surface area of Al/ZnO photocatalysts with different Al contents at 1%, 3%, 5% and 10% revealed the surface area of 63, 72, 89 and 68 m^2^/g, respectively. The specific surface area of all Al/ZnO NPs was higher than that of ZnO NPs. Thus, the Al doping can enhance a physical property of Al/ZnO NPs. It can be noted that the high specific surface area provides more active sites to the photocatalyst, contributing to the effectiveness of diffusion and reaction of MO dye molecules for photocatalytic degradation. 

**Figure 4:**
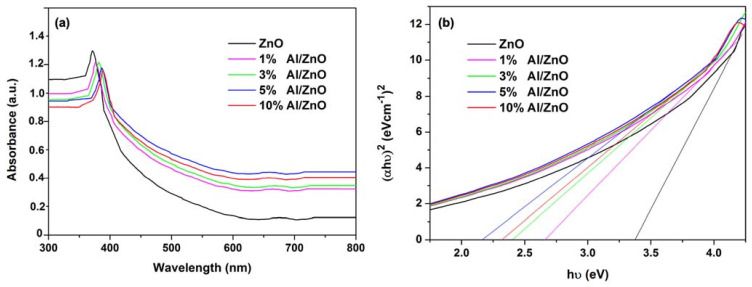
(**a**) Absorption spectra of ZnO and Al/ZnO photocatalysts analyzed by UV–Vis spectrophotometer; and (**b**) Tauc plots of energy band gap for ZnO and Al/ZnO photocatalysts.

The UV–Vis diffuse spectra of the pure ZnO and Al/ZnO photocatalysts with different Al contents are presented in Figure 4a. It can be seen that the Al/ZnO photocatalysts have a significant transition to visible area, compared to the undoped ZnO photocatalysts. The doping of Al metal and high structural homogeneity is the reason for the increased absorption of Al/ZnO NPs. The light absorption of Al/ZnO NPs increases for higher Al dosage in agreement with other studies [60,61]. The red shift to the longer wavelength of the absorption edges in visible light (400–700 nm) is observed on the Al/ZnO nanoparticles, which is expected to improve photocatalytic activity. The energy band gap of photocatalyst was determined using Tauc plot with the equation:

(4) $${\left(\propto h\upsilon \right)}^{n}=A\left(h\nu -E_{g}\right)$$

where α is absorption coefficient, *hυ* is photon energy and relation constant and *Eg* is optical band gap. In this study, the energy band gap of photocatalyst is calculated with the direct band gap n = 2. 

The calculated energy band gaps for the ZnO NPs with different Al contents of 1%, 3%, 5% and 10% are 3.34, 2.67, 2.40, 2.23 and 2.32 eV, respectively, as shown in Figure 4b. This result suggests that the bandgap energy of ZnO is changed to a lower level, which benefits the charge transfer from the valence band (VB) to the conduction band (CB). A similar trend was also reported by Mahdavi et al., who found that the shifted absorption edges were closely linked to the interaction of Al and ZnO, leading to the higher photocatalytic degradation efficiency [61]. 

**Figure 5:**
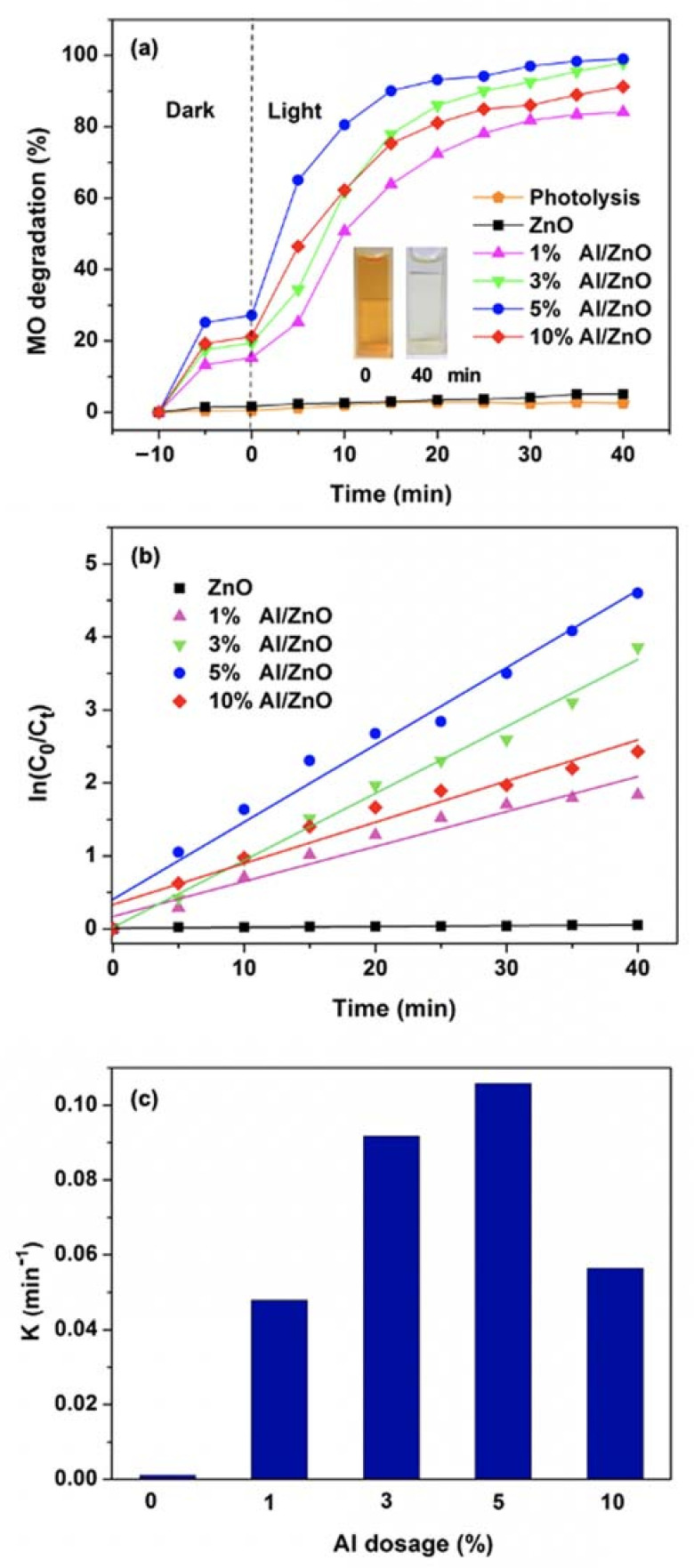
(**a**) Photodegradations (Inset shows the color change in MO at time intervals 0 and 40 min); (**b**) first−order reaction rates of MO dye; and (**c**) degradation rate constants of MO dye.

The photolytic of the MO dye and the photocatalytic activity of the Al/ZnO photocatalysts were determined by the decolorization of 50 mg/L MO dye solution under the visible irradiations, as shown in Figure 5a. The MO dye was barely degraded without photocatalyst, suggesting that the MO dye is stable and there is no photolysis of MO dye under visible light irradiation. It can be obviously observed that all Al/ZnO NPs exhibit quick adsorption in the dark. This could be attributed to the high specific surface area and small particle size of Al/ZnO NPs. It can be noted that the strong dark adsorption capacities of the Al/ZnO NPs improved their MO photocatalytic performances, in terms of MO decolorization and degradation processes. Furthermore, it was found that all the Al/ZnO photocatalysts provided better photocatalytic degradation performance than that of the pristine ZnO photocatalyst. These results demonstrate higher and faster photocatalytic degradation of MO with the Al/ZnO photocatalysts under visible light irradiation, compared to the photocatalytic performance of ZnO photocatalyst under the UV irradiation. 

This result also suggests that Al^3+^ ion can facilitate photocatalytic activity under the visible light region. The concentration of MO dye decreased gradually and saturated to the highest MO degradation rate within 40 min. Inset photo images in Figure 5a show the significant color change of the MO dye solution after 40 min photocatalytic reaction with Al/ZnO photocatalyst. The degradation efficiency of ZnO, 1% Al/ZnO, 3% Al/ZnO, 5% Al/ZnO and 10% Al/ZnO photocatalysts are 5%, 84%, 97%, 99% and 91%, respectively. It was found that the photocatalytic degradation efficiency of ZnO photocatalyst under UV light irradiation provided better performance than that of the ZnO photocatalyst under visible light irradiation. The lower photocatalytic efficiency of ZnO photocatalyst for MO degradation under visible light irradiation was due to its large bandgap, limiting the utilization of solar light to only UV light region. Furthermore, the 5% Al/ZnO photocatalyst exhibited the highest degradation efficiency, indicating that the optimum dosage of Al was 5%. Furthermore, the MO removal efficiency of 5% Al/ZnO NPs in this study is comparable to that of the Al/ZnO NPs reported by Mahdavi et al. [61], which was synthesized by sol-gel method. 

In Figure 5b, the time-dependent ln(C_0_/C) terms are illustrated for all Al/ZnO photocatalysts, according to first-order reaction kinetics. The calculated reaction rate constants (k) of the ZnO and Al/ZnO photocatalysts with different amounts of Al (1%, 3%, 5% and 10%) were 0.001, 0.048, 0.092, 0.106 and 0.056 min^−1^, respectively, as presented in Figure 5c. The reaction constants gradually increased with an increase in the Al dosage up to 5% which dropped thereafter. This photocatalyst have superior photodegradation efficiency under the visible light compared to the other photocatalysts that have been reported [47,50,60,61]. In addition, Al/ZnO photocatalysts have higher efficiency in degradation of MO compared to that of pristine ZnO, owing to the placement of Al at donor level to inhibit recombination of excited electrons and holes of valance band [60]. 

**Figure 6:**
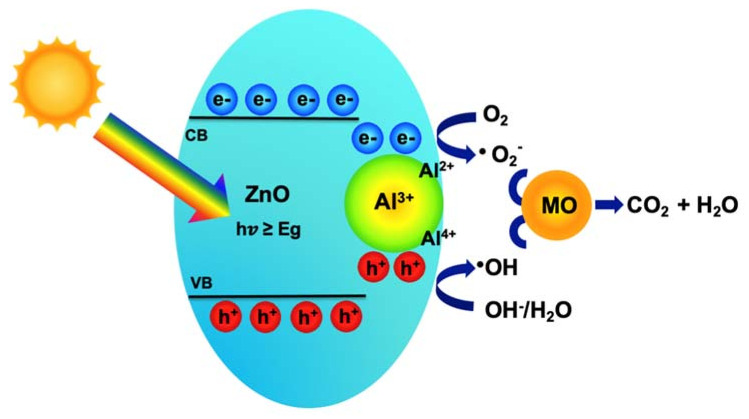
Mechanism of Al/ZnO photocatalyst for MO degradation under light irradiation.

The results of characterization and photocatalytic measurements demonstrate that the properties of ZnO photocatalysts strongly depend on the calcination temperature and Al dopant. The MO photocatalytic activity decreased gradually with the increase in calcination temperature. This can be attributed to the increased particle size and the reduced light absorption of the ZnO NPs at higher calcination temperatures. On the other hand, the enhancement of the MO photocatalytic activity was observed on the lower calcination temperature for ZnO NPs, further confirming the oxygen vacancies play a role in forming either active centers or trap centers [55]. In addition, the experimental results show that all modified ZnO photocatalysts with Al metal via a sol-gel method exhibited higher photocatalytic activity than the pristine ZnO photocatalyst. A schematic diagram of the photocatalytic degradation mechanism of MO dye molecules by the Al/ZnO photocatalysts under light irradiation is shown in Figure 6. Typically, the photons generated from irradiated light energy, equal or greater than the bandgap energy of ZnO, can activate the electrons in the valence band to the conduction band, leaving holes in the valence band. The photogenerated holes and electrons can then react with hydroxide (OH^-^) in the water or may directly oxidize the organic molecules and oxygen (O_2_) to generate active oxidizing species hydroxyl radicals (·OH) and superoxide radical (·O_2_^−^), respectively. Finally, the MO dye molecules are degraded into simpler inorganic molecules, CO_2_ and H_2_O by the active oxidizing species [61].

The improvement of the photocatalytic activity of Al/ZnO can be achieved by the Al doping effect on the energy bandgap of ZnO. The Al doping showed the significant photocatalytic degradation of MO, compared with the pristine ZnO. The complete mineralization of MO was achieved in only 40 min with 5% Al/ZnO, which could be explained by the reduction of bandgap energy of Al/ZnO, promoting charge generation efficiency of photogenerated electrons under visible light irradiation. Moreover, the introduction of Al metal can prevent recombination of charge carriers that play the trap role. The Al^3+^ ions trap and react with electrons and holes, then generate Al^2+^ and Al^4+^ ions, respectively. The Al^2+^ and Al^4+^ ions further react with O_2_ and OH^−^ to create ·O_2_^−^ and ·OH ^−^, respectively, on the photocatalyst surface [50,62]. Hence, the enhanced photocatalytic degradation performance of the Al/ZnO photocatalysts should be attributed to the effectiveness of charge generation, charge separation and charge transfer by Al elements under visible light irradiation. The relevant reaction mechanism is shown as follows:

ZnO + *hv* → e^−^ + h^+^

Al^3+^ + e^−^ → Al^2+^

Al^2+^ + O_2_ → · O_2_^−^ + Al^3+^

Al^4+^ + OH^−^ → ·OH + Al^3+^

Al^3+^ + h^+^ → Al^4+^

h^+^ + OH^−^/H_2_O → ·OH

e^−^ + O_2_ → ·O_2_^−^

·OH/·O_2_^−^ + MO dye → CO_2_ + H_2_O

Under visible light irradiation, the main degradation pathway is closely related with the formation of radicals by charge migration to decompose the dye molecules. It could be stated here that Al plays the synergistic role by reducing the bandgap energy and promoting the charge separation of Al/ZnO. It is in good agreement with the study by Saber et al. [63] who proposed that the improved photocatalytic activity of Al/ZnO NPs in decomposing naphthol green B was mainly attributed to the bandgap reduction and the extra electrons by doping Al. Similarly, Lee et al. [64] reported Al-doped ZnO NPs with different Al concentrations for the photocatalytic degradation of MO. The photocatalytic degradation of MO increased with the increased Al concentration and its reaction rate constant showed a maximum at 3% Al. However, the doping of Al into ZnO did not significantly change the light absorption property of ZnO. The enhanced photocatalytic activity of Al/doped ZnO NPs could be attributed to effective suppression of photoinduced electrons and holes, while an excess amount of Al could act as the recombination center for photogenerated electron–hole pairs, resulting in the adverse effect on the photocatalytic activity of Al/ZnO.

## 4. Conclusions

ZnO NPs and visible-light responsive Al-doped ZnO NPs were achieved via the facile sol-gel method at low calcination temperature. The calcination temperatures and Al dopant indicated significant effects on morphology, absorption property and photocatalytic activity of ZnO NPs. FE-SEM analysis revealed the transformation of the sphere-like particles into nanorods and nanoflake upon the variation of calcination temperatures (200, 300 and 400 °C) for ZnO NPs. However, XRD analysis confirmed that these calcined ZnO NPs presented a well-crystalline hexagonal wurtzite structure. The ZnO NPs calcined at 200 °C revealed a uniform spherical structure with superior properties, including a good optical property, high specific surface area and excellent photocatalytic activity in the UV light irradiation. With an increment of Al dopant, the Al/ZnO NPs demonstrated the red shifting of the absorption spectrum, indicating the narrower bandgap which leads to excellent and rapid photocatalytic activity compared with the undoped ZnO NPs. Importantly, Al metal dopants played a key role in extending the light absorption from UV region to visible region with suppression of the charge recombination and the formation of hydroxyl groups on catalyst surfaces. Doping 5% Al on ZnO NPs was an optimum dosage to enhance photocatalytic degradation efficiency of MO. Notably, photocatalytic activity of ZnO and Al/ZnO NPs significantly depended on calcination temperature, doped metal ion nature and its concentration. It is expected that the Al/ZnO photocatalyst can be a promising candidate as a low-cost and environmentally-friendly photocatalyst system for high performance wastewater treatment applications.

## Data Availability

Not applicable.
